# Predictors of adverse maternal and perinatal outcomes in a refugee population from an active conflict country, Syria

**DOI:** 10.4274/tjod.galenos.2019.98752

**Published:** 2019-10-10

**Authors:** Serap Fırtına Tuncer, Burcu Timur, Ethem Serdar Yalvaç, Leyla Mollamahmutoğlu

**Affiliations:** 1 University of Health Sciences, Etlik Zübeyde Hanım Women’s Diseases Training and Research Hospital, Clinic of Obsttetrics and Gynecology, Ankara, Turkey

**Keywords:** Refugees, Syrian, immigrants, pregnancy, perinatal outcome, maternal outcome

## Abstract

**Objective:**

To elucidate predictors of adverse maternal and perinatal outcomes in refugees emigrating from an active conflict region (Syria).

**Materials and Methods:**

This study included Syrian pregnant women who gave birth in Etlik Zübeyde Hanım Training and Research Hospital between 2013 and 2016. Adverse perinatal outcomes were defined as preterm labor, premature rupture of membranes, early membrane rupture, intrauterine growth retardation, hypertension, perinatal excites, and erythrocyte-transfused cases. Factors associated with those adverse outcomes were assessed using multiple logistic regression analysis.

**Results:**

Having an active smoking habit [odds ratio (OR): 2.647, 95% confidence interval (CI): 1.767-3.965; p<0.001], obesity (OR: 2.272, 95% CI: 1.396-3.699; p=0.001), and adolescent age (OR: 1.732, 95% CI: 1.204-2.491; p=0.003) were found to be the most important predictors of adverse maternal and perinatal outcomes. Eighty of 129 (62%) smokers, 45 of 81 (55.65%) obese individuals, and 91 of 169 adolescents (53.8%) had adverse maternal and perinatal outcomes.

**Conclusion:**

Prevention strategies for obesity, smoking, and adolescent pregnancies should be implemented primarily to reduce maternal and antenatal adverse outcomes. Pregnant women with these risk factors in a refugee community emigrating from a conflict-zone nation should be followed up closely.

**PRECIS:** Smoking, obesity, and adolescence are the most important predictors for adverse maternal and perinatal outcomes in pregnant refugees.

## Introduction

Refugees, especially those emigrating from their home country due to war or other hardships, face many difficulties in their new host countries. Changes in food consumption, economic difficulties, language barriers, and limitations in accessing healthcare may disturb their health-related conditions^(1,2,3)^. Being a vulnerable group, immigrant pregnant women in particular experience more difficulties and perhaps may have more adverse outcomes. Since March 2011, the Turkish government has begun hosting millions of Syrian refugees, preparing refugee camps and giving them the opportunity to travel to all cities of the country. At present, more than 3.5 million Syrian refugees are scattered all around Turkey^(4)^. According to the United Nations Refugee Agency, Turkey is the most accessed hosting country for refugees worldwide^(4)^. Previous studies have shown an increased risk of adverse maternal and perinatal outcomes for emigrating pregnant women including maternal anemia, preterm birth, low birth weight, bleeding during delivery, psychological distress, perineal laceration, and postpartum hemorrhage^(1,2,3,5,6,7)^. Besides these data, pregnant women leaving conflict regions have increased adverse perinatal complications as compared with pregnant women from non-conflict regions^(8,9)^. A pregnant refugee’s country of origin is found to be an important predictor of pregnancy outcomes. Moreover, immigration itself has a negative impact on pregnancy outcomes in the same nation^(10,11)^.

Predictors of adverse events in pregnancy in among women emigrating from a nation in an active conflict region have not been well studied to date. Therefore, in the present study, we sought to identify predictors of adverse maternal and perinatal outcomes in refugees coming from an active conflict region, specifically Syria.

## Materials and Methods

### Study design

The maternity clinic at Etlik Zübeyde Hanım Training and Research Hospital is a tertiary maternity unit with a neonatal intensive care unit (NICU). Most patients seen here, including both refugees and Turkish people, are referred from other clinics when gestational and fetal complications are suspected. For this study, we employed a Syrian interpreter to communicate with the refugees. After approval of the study protocol by our institutional review board, the files and birth records of Syrian refugees concerning the period 2013 through 2016 were retrospectively analyzed. As the Turkish government provided a translator, hospital staff and medical team communicated with the patients with the help of a translator or relatives speaking Turkish. To establish a high-quality dataset, only parameters of singleton pregnancies with complete data were included. Parameters including gestational diabetes mellitus and systemic disorders were not included in the analysis because the refugee population had poor antenatal care. All patients were evaluated using ultrasonography at the time of delivery and according to need at each other visit. Demographic data including maternal age, gravidity, parity, smoking habits, history of abortion, route of delivery in previous births, gestational age at delivery, number of antenatal follow-ups, mode of delivery, and maternal hemoglobin (Hb) levels were extracted. Patients’ body mass index (BMIs) were calculated based on their height and body weight at the time of admission to hospital for delivery. Obese patients were defined as those with BMI values equal to or greater than 30 kg/m^2^. Adverse perinatal outcomes were defined as preterm labor, premature rupture of membranes (PPROM), early membrane rupture (EMR), intrauterine growth retardation (IUGR), hypertension, perinatal exitus, and erythrocyte-transfused cases. The newborn’s birth weight, one and five-minute Apgar scores, heart pulse rates, grimace response, activity, respiration scores, admission to the NICU were recorded. Gestational age was determined based on the first day of the last menstruation period (LMP) of the mother. For patients who did not know their LMP, gestational age was calculated based on their earliest obstetric ultrasound. Hypertension during pregnancy was defined as a blood pressure of more than 140/90 mmHg measured on two occasions. Preterm delivery was defined as birth before 37 weeks, and IUGR was determined based on clinical evaluation and ultrasonographic parameters. Adolescent pregnancy was noted when the patient was younger than 20 years of age at the time of delivery.

### Statistical Analysis

The Statistical Package for the Social Sciences version 23.0 for Windows (IBM Corp., Armonk, NY, USA) software package was used and p values less than 0.05 were defined as statistically significant. Variables with p values less than 0.1 on univariate analysis were included in binominal logistic regression analysis.

## Results

A total of 747 pregnant Syrian refugees had deliveries between 2013 and 2016. Eight twin pregnancies and 35 patients with incomplete data were excluded from this study. Therefore, a total of 698 Syrian refugees were included in the present study, including 167 adolescents (23.9%) aged between 12 and 19 years. The clinical characteristics of the study population are presented in Table 1. The median age of the study participants was 24 years, and the majority (67.2%) were multigravida. Most patients (n=370) had visits only during the third trimester (53.0%), and only 220 (31.5%) patients had four or more antenatal visits. Adverse maternal and perinatal outcomes were present in 306 (43.8%) cases. In 83 patients, multiple adverse maternal and/or perinatal outcomes were recorded as follows: preterm labor was observed in 134, IUGR in 86, and PPROM or EMR in 81, respectively. Additionally, gestational hypertension in two out of 11 women resulted in ablatio placenta, intrauterine exitus was seen in seven, neonatal exitus was observed in eight, and severe maternal anemia requiring erythrocyte transfusion was present in 45 women (Table 2). Table 3 demonstrates the relationship between adverse maternal-fetal outcomes and possible risk factors. Adolescent pregnancy (p=0.003), being a smoker (p<0.001), obesity (p=0.024), and a number of antenatal visits of less than four (p<0.001) were related with adverse maternal and fetal outcomes.

### Results of logistic regression analysis

The probability of adverse maternal and perinatal outcomes increased 2.647 times [95% confidence interval (CI) for odds ratio (OR): 1.767-3.965] in smokers when compared with nonsmokers (p<0.001). Obesity (BMI >30 kg/m^2^) increased the probability of adverse maternal-fetal outcomes 2.272 times (95% CI for OR: 1.396-3.699) relative to patients with BMIs of 30 k/m^2^ or less (p=0.001). Also, the probability of adverse maternal-fetal outcomes increased 1.732 times (95% CI for OR: 1.204-2.491) in adolescent pregnant women in comparison with adult pregnant women (p=0.03). In the regression analysis, gravidity of more than two and less than four antenatal visits were not significant indicators of adverse maternal-fetal outcomes (Table 4). Correlations between the detected risk factors and each adverse maternal or perinatal outcome are presented in Table 5. Maternal anemia requiring erythrocyte transfusion was found as a more common feature of nonsmoking pregnant women versus those with an active smoking habit (p=0.008). Preterm birth (p=0.022), PPROM or EMR (p<0.001), IUGR (p<0.001), and perinatal exitus (p<0.001) were more commonly observed in smokers than in nonsmokers. PPROM or EMR (p=0.039) and hypertension (p=0.009) were additionally found more commonly in obese patients versus non-obese patients. Lastly, preterm birth (p=0.018) and IUGR (p<0.001) were more commonly observed in adolescent pregnant women than in adult pregnant women.

## Discussion

This study showed that an active smoking habit [OR: 2.647, 95% CI: (1.767-3.965 9); p<0.001], obesity [OR: 2.272, 95% CI: (1.396-3.699); p=0.001], and adolescence [OR: 1.732, 95% CI: (1.204-2.491); p=0.003] were the most important predictors for adverse perinatal outcomes of pregnant women emigrating from an active war region. Eighty of 129 (62%) smokers, 45 of 81 (55.65%) obese individuals (BMI >30 kg/m^2^), and 91 of 169 (53.8%) adolescent pregnant women had any one of the following adverse maternal and perinatal outcomes: preterm birth, PPROM, EMR, IUGR, hypertension, a need for erythrocyte transfusion or perinatal exitus. As compared with nonsmokers, smoking during pregnancy increased the risks of preterm birth [OR: 2.6, 95% CI: (1.1-1.6) for >10 cigarettes/day], and PPROM [OR: 1.97, 95% CI: (1.32-2.94) for >10 cigarettes/day] depending on the pack/years of smoking^(12,13)^. A meta-analysis of 46 studies documented that any amount of smoking increased the risk of perinatal death (OR: 1.33, 95% CI: 1.25-1.41)^(14)^. Besides, smoking had a negative impact on fetal growth and increased the risk of IUGR (OR: 2.07, 95% CI: 1.69±2.53)^(15)^. In accordance with previous studies, we found that, even in a migrant population, smoking increased the risks of preterm birth, PPROM, IUGR, and perinatal death (Table 4). It was reported that anemia is not a generalized sign, and elevated Hb levels were detected in smokers^(16)^. Accordingly, we found that a need for blood transfusion for anemia was more frequent among nonsmokers in the refugee population. Obesity is the principle health problem that causes adverse effects in pregnancy^(17)^. Obesity is related with various adverse obstetric complications such as gestational diabetes mellitus, hypertension, venous thromboembolism, shoulder dystocia, prematurity, stillbirth, neonatal death, postpartum hemorrhage, and urinary, uterine, and wound infections^(18)^. A prevalence rate of 46.4% was reported in a study among Syrian women in 2006^(19)^. In the present study of refugee women, we found obesity at a rate of 11.6%, which is significantly lower than that reported in the literature. This may be explained by the negative impact of war on adequate nutrition and its related factors (e.g., difficulty in reaching food, low socioeconomic levels, and increased psychological problems such as depression). In the present study, we found that obesity was not associated with idiopathic preterm birth; this finding was in accordance with the outcomes of a study of nearly 1.600.000 deliveries in a population-based cohort study of women with live singleton births in Sweden^(20)^. Cnattingius et al. ^(20)^ showed that obesity increased the incidence of preterm births, especially in the context of deliveries happening between 22 and 27 gestational weeks. In their study, the authors stated that obesity was related with preterm delivery because of PPROM and other obesity-related diseases such as hypertension and diabetes; when these conditions were excluded, the preterm delivery risk was unchanged in obese patients^(20)^. Similarly, in a series of 4653 preterm births among more than 55.000 births, Madan et al. ^(21)^ also stated the adverse impact of diabetes and hypertension on the relationship between obesity and premature birth. In accordance with these prior studies, we found that obesity was not related with idiopathic preterm birth but was with hypertension and PPROM (Table 4). Indeed, Zhong et al.^(22)^ showed that obesity was associated with a decreased risk of spontaneous preterm birth without PPROM at less than 37 weeks’ gestation (OR: 0.8, 95% CI: 0.7-0.9) and increased risks of PPROM before 37 and 34 weeks’ gestation [OR: 1.3, 95% CI: (1.1-1.6) and OR: 1.4, 95% CI: (1.0-2.0), respectively]. Adolescent pregnancy is one of the major health problems in refugee populations, resulting in adverse maternal-fetal outcomes. In a multinational study organized by the World Health Organization, Ganchimeg et al.^(23) ^documented more adverse maternal and adverse fetal outcomes among adolescent mothers (aged ≤20 years) than among mothers aged 20 to 24 years. It is suggested that significantly younger adolescent mothers carry an especially higher risk of preterm delivery. In other words, mothers aged 15 years or younger are 1.6 times more likely to experience preterm delivery as compared with mothers aged 20 to 24 years (OR: 1.60, 95% CI: 1.37-1.87). In the study by Ganchimeg et al.^(23)^ 11.2% of mothers aged 10 to 15 years, but only 7% of mothers aged 20 to 24 years had a risk of preterm delivery (p<0.001). In our study, we found that 25.4% of adolescent and 17.2% of adult mothers had a risk of preterm delivery^(23)^. Besides this, we also documented a higher risk of IUGR in the adolescent group (21.9%) as compared with the adult group (9.3%) (p<0.001). Although we did not have the opportunity to compare the adult refugee population with an adolescent control group, we estimated higher risks of adverse maternal and perinatal outcomes in an adolescent refugee population as a result of the insufficient food supply, higher workloads, and malnutrition.

### Study Limitations

There are some limitations to our study. Regression analysis results revealed 46% sensitivity and 74% specificity rates for the estimation of the risk of adverse maternal and perinatal outcomes. To increase the sensitivity and specificity of calculations, some other possible risk factors could be added to the study. However, this was a retrospective study, making it impossible to adequately evaluate all possible factors related to adverse outcomes in pregnancy. Additionally, our sample size was not large enough to be sufficiently powered to examine the relationship between possible risk factors and rates of pregnancy complications due to the low prevalence rates of stillbirth, preeclampsia, congenital anomalies, venous thromboembolism, and maternal death. Secondly, most of the patients in our series were referred from other clinics when a high-risk pregnancy complication was suspected, potentially introducing bias into the patient selection scheme for this study. Despite these limitations, our study is unique due to its evaluation of risk factors for adverse maternal and perinatal outcomes in a refugee population coming from an active conflict country. We believe that taking the results of this study into account could help in reducing adverse outcomes in pregnant refugee populations.

## Conclusion

This study showed that smoking, obesity, and adolescence were the most important predictors for adverse maternal and perinatal outcomes in pregnant refugees coming from an active conflict region. Prevention strategies for obesity, smoking, and adolescent pregnancies should be implemented, primarily to reduce maternal and antenatal adverse outcomes. Additionally, pregnant women with these risk factors in a refugee communities emigrating from nations in conflict zones should be followed up closely.

## Figures and Tables

**Table 1:**
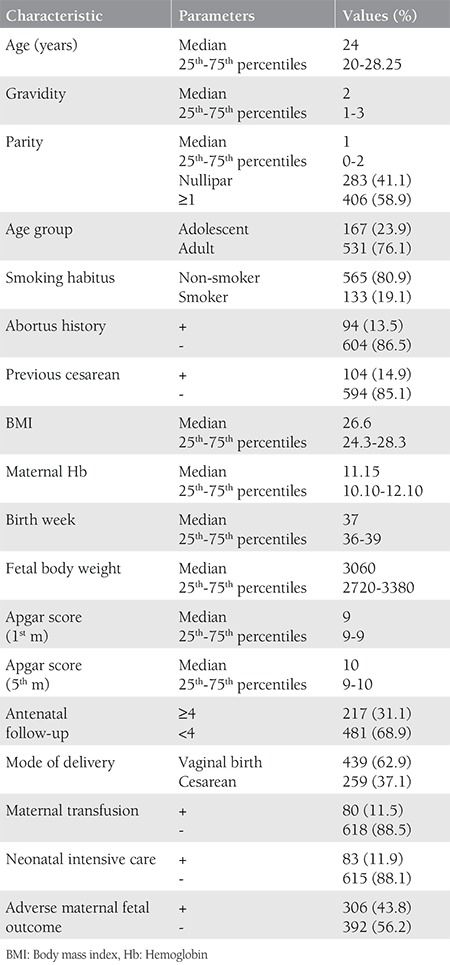
Characteristics of study population

**Table 2:**
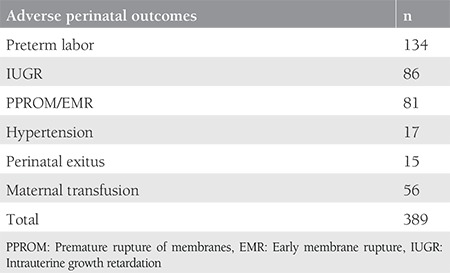
Number of adverse maternal and perinatal outcomes

**Table 3:**
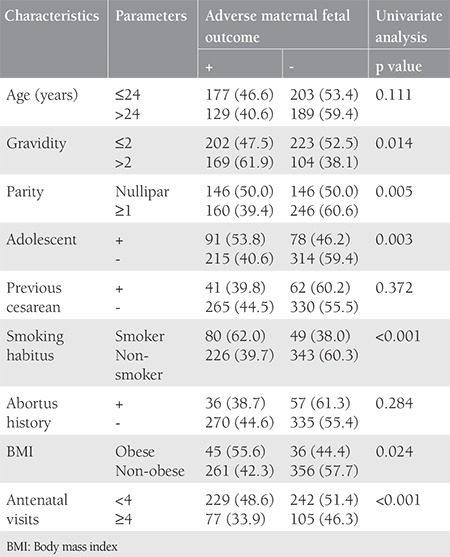
Relationship between adverse maternal perinatal outcome and possible risk factors

**Table 4:**
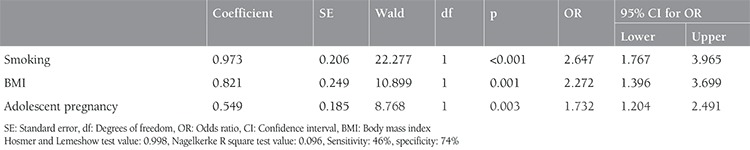
Logistic regression results of the risk factors for adverse maternal and fetal outcomes

**Table 5:**
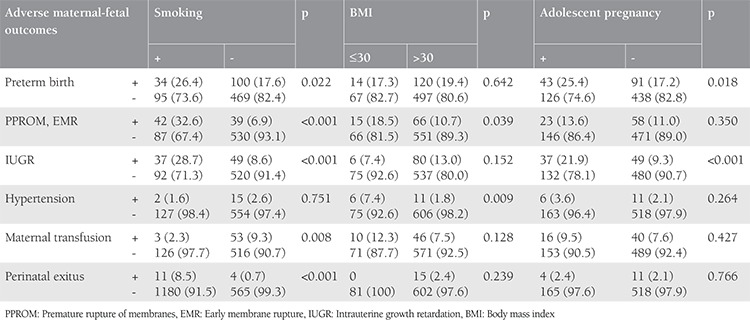
Relationship between risk factors and adverse maternal-perinatal outcomes
